# Acute Measles Encephalitis in Partially Vaccinated Adults

**DOI:** 10.1371/journal.pone.0071671

**Published:** 2013-08-13

**Authors:** Annette Fox, Than Manh Hung, Heiman Wertheim, Le Nguyen Minh Hoa, Angela Vincent, Bethan Lang, Patrick Waters, Nguyen Hong Ha, Nguyen Vu Trung, Jeremy Farrar, Nguyen Van Kinh, Peter Horby

**Affiliations:** 1 Oxford University Clinical Research Unit, Wellcome Trust Major Overseas Programme, Ho Chi Minh City and Ha Noi, Viet Nam; 2 Centre for Tropical Medicine, Nuffield Department of Clinical Medicine, University of Oxford, Oxford, United Kingdom; 3 National Hospital for Tropical Diseases, Ha Noi, Viet Nam; 4 Neurosciences Group, Department of Clinical Neurology, University of Oxford, John Radcliffe Hospital, Oxford, United Kingdom; The George Washington University Medical Center, United States of America

## Abstract

**Background:**

The pathogenesis of acute measles encephalitis (AME) is poorly understood. Treatment with immune-modulators is based on theories that post-infectious autoimmune responses cause demyelination. The clinical course and immunological parameters of AME were examined during an outbreak in Vietnam.

**Methods and Findings:**

Fifteen measles IgM-positive patients with confusion or Glasgow Coma Scale (GCS) score below 13, and thirteen with uncomplicated measles were enrolled from 2008–2010. Standardized clinical exams were performed and blood collected for lymphocyte and measles- and auto-antibody analysis. The median age of AME patients was 21 years, similar to controls. Eleven reported receiving measles vaccination when aged one year. Confusion developed a median of 4 days after rash. Six patients had GCS <8 and four required mechanical ventilation. CSF showed pleocytosis (64%) and proteinorrhachia (71%) but measles virus RNA was not detected. MRI revealed bilateral lesions in the cerebellum and brain stem in some patients. Most received dexamethasone +/− IVIG within 4 days of admission but symptoms persisted for ≥3 weeks in five. The concentration of voltage gated calcium channel-complex-reactive antibodies was 900 pM in one patient, and declined to 609 pM ∼ 3 months later. Measles-reactive IgG antibody avidity was high in AME patients born after vaccine coverage exceeded 50% (∼ 25 years earlier). AME patients had low CD4 (218/µl, p = 0.029) and CD8 (200/µl, p = 0.012) T-cell counts compared to controls.

**Conclusion:**

Young adults presenting with AME in Vietnam reported a history of one prior measles immunization, and those aged <25 years had high measles-reactive IgG avidity indicative of prior vaccination. This suggests that one-dose measles immunization is not sufficient to prevent AME in young adults and reinforces the importance of maintaining high coverage with a two-dose measles immunization schedule. Treatment with corticosteroids and IVIG is common practice, and should be assessed in randomized clinical trials.

## Introduction

Measles is a highly contagious vaccine preventable illness. Globally, measles deaths fell by 50% between 2000 and 2008 following intensified vaccination campaigns but recent large outbreaks demonstrate the potential for rapid recrudescence in under-immunized groups [1]. Around 30% of reported measles cases develop complications [2]. Pneumonia is the commonest complication followed by acute measles encephalitis (AME), which occurs in 1–3 per 1000 infected persons and is more common in adults than children [2]. Other serious CNS complications are rare in immune competent people [3]. Sub-acute sclerosing panencephalitis (SSPE) occurs in ∼ 1 per 10000 infected persons many years after the initial infection and is the result of persistent measles virus infection of the CNS [4]–[5].

The clinical course and pathogenesis of AME are not well understood. Although lymphoid cells are the principle targets for measles virus infection, measles can infect neurons [6]–[7], and appears to have several mechanisms for circumventing the blood brain barrier [8]–[9]. Recent studies also indicate that CNS infection may be relatively common, with measles virus RNA detected by RT-PCR at autopsy in the brain of around 19% of individuals that never had CNS disease [7], [10]. T cell responses control neurological infection in mouse models [11] and may therefore be an important factor in preventing encephalitis in humans. However, AME is often termed measles post-infectious encephalitis or PIE because symptoms generally start 3–10 days after the onset of rash [2]. In addition, measles virus has rarely been detected in post-mortem CNS tissue collected at the time of acute encephalitis using immunohistochemistry, which may be less sensitive than RT-PCR but detects measles virus in post-mortem brain tissue from fatal SSPE [6], [12]. Myelin basic protein has been detected in cerebrospinal fluid (CSF) and nearly 50% of patients have lymphocyte proliferative responses to myelin basic protein [13]. Consequently it has been proposed that AME is an immune-mediated demyelinating syndrome [6], [12]–[15]. The role of myelin reactive autoantibodies is controversial [16]–[17]. In animal models injection of myelin leads to the production of myelin-reactive antibodies and pathology similar to that in AME, but techniques such as ELISA and radioimmunoassay fail to detect any increase in myelin-reactive antibody in patients with CNS pathology [17]. Recently assays that detect conformation sensitive myelin-reactive antibodies have detected increased levels in a subset of acute disseminated encephalomyelitis patients [16]. Immune-modulators including intravenous immunoglobulin (IVIG) [18]–[20] corticosteroids [20]–[22] and plasmapheresis [23] have been used to treat AME, but with variable effect.

Measles vaccination was introduced in Vietnam in 1982 [24] and coverage with one-dose at one year of age has exceeded 50% since 1986 and 90% since 1993 [25]. However, a measles outbreak started in Northern Vietnam in October 2008. In contrast to the pre-vaccine era, incidence was high in 20–24 year olds [24]–[25] and a number of cases of adult AME were admitted to the National Hospital of Tropical Diseases (NHTD). Paradoxically, the epidemiology partially reflects the success of vaccination in reducing transmission and childhood illness because there is less natural infection to boost vaccine-induced immunity such that protection starts to wane in young adults [24]–[25]. This has been widely recognized and a two-dose vaccine schedule is now recommended and was adopted in Vietnam in 2006 [24]. Patients admitted to NHTD with measles AME were treated with intravenous immunoglobulin (IVIG) and corticosteroids. A prospective observational study was rapidly instigated to describe the clinical and neurological course of illness in relationship to treatment. To investigate the mechanisms underlying AME pathogenesis a case-control study was also initiated in which blood samples were collected from AME patients and measles patients without CNS involvement for immune phenotyping, autoantibody detection and measles serology.

## Methods

### Ethics Statement

All participants provided written informed consent. The study was approved by the ethical review boards of The National Hospital for Tropical Diseases (NHTD), Vietnam and the Oxford Tropical Research Ethics Committee, University of Oxford, UK (reference 06 09).

### Patient Recruitment and Clinical Investigation

Patients admitted to NHTD with fever and rash in the preceding fortnight, measles-reactive IgM in sera, and confusion or a Glasgow Coma Scale (GCS) score <13 were asked to participate. The GCS is an objective measurement of level of consciousness based on eye opening, verbal performance and motor response. The main relevant disadvantages of the GCS in terms of the current study are the inability to assess the verbal score in intubated patients, and the exclusion of brainstem reflex assessment. The GCS score ranges from 15 to 3 representing the most and least responsive, respectively. Values >12 have been associated with good neurological outcome whereas values ≤8 predict a poor outcome [26]. The NHTD is a tertiary care centre for adults with infectious diseases in Hanoi that also serves as a referral centre for infectious diseases in Northern Vietnam. AME patients were assessed on days 0, 7 and 14 of admission, at discharge, and at 3 and 12-months post-discharge by a team of doctors with experience in measles diagnosis and treatment. Clinical and neurological findings were prospectively recorded using standardized case record forms (CRFs). Peripheral blood was also collected for serology and immunology. CSF was collected when clinically indicated. Patients with fever, rash and measles-reactive IgM but GCS ≥13 and no confusion were asked to participate as controls and were prospectively assessed at admission only. Subsequently, patient folders were reviewed and relevant clinical information included.

### Laboratory Investigations

Measles-reactive IgM antibodies were detected in sera using PLATELIA™ Measles IgM ELISA kit (Bio-Rad Marnes-la-Coquette France), performed according to the manufacturers instructions. A real-time RT-PCR developed by Mubarak et al [27] was used to detect measles virus genomic RNA in sera, CSF and throat swabs. The primers amplify a nucleoprotein gene fragment and the limit of detection is 2 cell culture infectious dose 50% units (CCID50) [27]. Radioimmunoassay was used to determine serum and CSF levels of antibodies against voltage gated potassium channel-complex (VGKC) [28], voltage gated calcium channels (VGCC), and glutamic acid decarboxylase (GAD) as described previously [29]. A cell-based assay was used to detect antibodies to N-methyl-D-aspartate (NMDA) receptors [30]. To detect myelin oligodendrocyte (MOG) antibodies human embryonic kidney cells on coverslips were transiently transfected with full length human MOG for 40 hrs. Cells were incubated for 1 hr with sera diluted 1∶20, fixed in 3% formaldehyde then incubated with goat anti-human IgG Alexa fluor 488 (1∶500; Invitrogen) for 1 hr. After washing, coverslips were mounted onto slides and fluorescence scored from 0 (no signal) to 4 (very bright surface fluorescence) with scores of 1 or greater considered positive. Measles virus IgG titer and avidity were determined using a commercial ELISA kit (Euroimmun, D-23560 Lubeck, Germany) performed according to the manufacturer’s protocols. Samples were diluted appropriately to fall within the standard range (5–5000 U/ml) and were tested with and without addition of urea. Absolute cell counts were performed on EDTA venous blood within 8 hours of collection using a lyse no-wash procedure with Multitest 6-color TBNK Reagent and TruCount tubes, and analysis on a FACSCanto (BD Biosciences, San Jose (CA), USA), as described previously [31]. Cut-offs for identifying lymphopenia and lymphocyte subset penia were derived from values for recovered patients assessed during follow-up visits in this and other studies of influenza and dengue patients, as described previously [31].

### Analysis

Data were double entered into an Access database. Illness day was calculated from the date fever commenced, which was assigned as day 0. Proportions were compared using odds ratios and Chi-Square or Fisher’s exact test when any expected cell count was less than 5. Continuous variables were presented as median and interdecile range (IDR) and compared using Mann Whitney tests. IgG avidity cut-offs for classifying infections as primary (OD with urea <40% of untreated OD) or secondary (OD with urea >60% of untreated OD) were as recommended by the manufacturer. High and low avidity controls provided fell within appropriate cut-offs. A number of other studies have used urea-based IgG avidity tests with similar cut-offs to differentiate between primary infection and secondary vaccine failure or chronic infection as in SSPE [32]–[33]. Linear regression was used to assess the dependence of measles-reactive IgG concentration and avidity on and age and day since fever onset. Analyses were performed with SPSS for Windows, Rel. 14.0.0.245, 2005 (SPSS Inc. Chicago (IL), USA).

## Results

### Demographics and Clinical Presentation

Fifteen AME patients were enrolled between December 2008 and May 2010. Their median age was 21 years (IDR 19–37 years), with only one born before 1984. Ten (67%) were male, eleven (92%) were students and eight (53%) were from Hanoi. The remaining seven were from five other northern and central provinces. Eleven (73%) reported being vaccinated at 1 year of age; two reported they had not been vaccinated and two did not know. In most cases a parent was asked about the patient’s vaccination status but vaccination cards were not seen. Fourteen were previously healthy and one (#031) had a history of glomerulonephritis. Sixteen patients admitted during the same time interval with fever and rash were enrolled as controls. Subsequently, one was found to have had encephalitis symptoms and two were measles IgM negative, and were excluded. The thirteen controls included in the analysis developed only severe headache and fatigue, none had pneumonia. Their median age was 23 years (IDR 19–31 years), 5 were born before 1984, and five (38%) were males. Only five controls were asked about their vaccination status, two aged 28 said they had not been vaccinated, two aged 19 and 21 said they had been vaccinated at 1 year of age, and one did not know.

**Figure 1:**
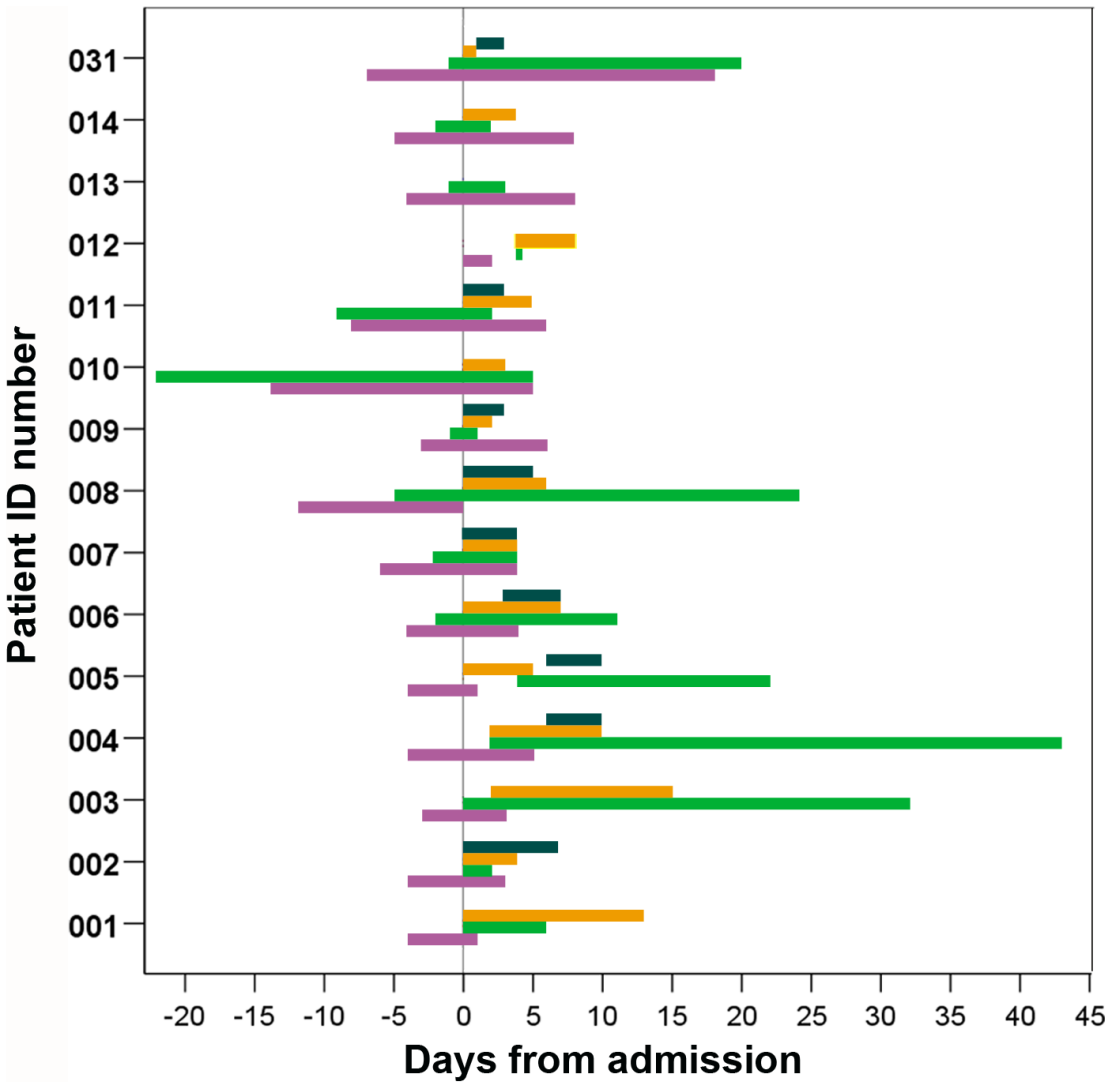
Timing of confusion in relation to rash and treatment with steroids and IVIG for 15 AME patients. Rash (purple); confusion (green); steroids (orange); IVIG (dark green).

All AME patients presented with a history of rash as per the inclusion criteria. Other typical measles symptoms were also common (Table 1) including cough (93%), headache (87%) and sore throat (60%). Forty seven percent had conjunctivitis, 33% had diarrhea and/or vomiting and two had lymphadenopathy on or prior to admission (Table 1). Admission pulse was elevated compared to recovery pulse in eleven patients. Confusion started 6 (0–10) days after fever and 4 (−3.8–7.4) days after rash (Figure 1). GCS scores at admission were in the severe range (≤8) for two patients, in the moderate range (9–12) for nine patients and mild or normal (13–15) for four patients (Table 1). Four patients presented with convulsions, four with neck stiffness, three with agitation and one with coma (Table 1).

**Table 1:** Presentation signs and symptoms of 15 measles acute encephalitis patients.

**Patient ID**	001	002	003	004†	005	006	007	008	009	010	011	012†	013	014	031
**Age**	37	20	22	25	19	20	21	21	24	23	19	25	19	24	20
**Gender**	M	M	M	M	F	M	M	M	F	M	F	F	M	F	M
**Vaccinated**	**+**	**+**	**–**	?	?	**+**	**–**	**+**	**+**	**+**	**+**	**+**	**+**	**+**	**+**
**Days febrile**	7	6	6	5	7	6	8	15	5	19	11	3	4	7	7
**Cough**	**+**	**+**	**+**	**–**	**+**	**+**	**+**	**+**	**+**	**+**	**+**	**+**	**+**	**+**	**+**
**Headache**	**+**	**+**	**+**	**–**	**+**	**+**	**+**	**+**	**+**	**+**	**+**	**+**	**+**	**+**	**+**
**Sore throat**	**–**	**+**	**–**	**–**	**–**	**+**	**–**	**+**	**+**	**+**	**+**	**+**	**+**	**+**	**–**
**Conjunctivitis**	**–**	**+**	**–**	**–**	**+**	**+**	**–**	**+**	**+**	**–**	**+**	**+**	**–**	**–**	**–**
**Diarrhea**	**–**	**–**	**+**	**–**	**–**	**–**	**–**	**+**	**+**	**–**	**–**	**–**	**+**	**–**	**–**
**Vomiting**	**–**	**–**	**+**	**–**	**–**	**–**	**–**	**+**	**+**	**–**	**–**	**–**	**–**	**+**	**–**
**Pulse**	90	110	95	90	100	90	118	90	110	100	85	90	90	90	100
**GCS**	13	12	10	15	8	11	8	11	10	13	10	15	10	10	10
**Confusion**	**+**	**+**	**+**	**–**	**–**	**+**	**+**	**+**	**+**	**+**	**+**	**–**	**+**	**+**	**+**
**Convulsion**	**+**	**+**	**–**	**–**	**–**	**–**	**–**	**–**	**–**	**+**	**+**	**–**	**–**	**–**	**–**
**Agitation**	**–**	**–**	**+**	**–**	**–**	**–**	**–**	**+**	**+**	**–**	**–**	**–**	**–**	**–**	**–**
**Neck stiffness**	**–**	**+**	**+**	**+**	**–**	**–**	**–**	**–**	**–**	**–**	**–**	**–**	**–**	**–**	**+**
**Limb weakness**	**–**	**–**	**–**	**–**	**–**	**–**	**–**	**–**	**–**	**–**	**–**	**–**	**–**	**–**	**–**
**Other symptoms** §	**–**	**–**	**–**	**–**	**–**	**+**	**–**	**+**	**–**	**–**	**+**	**–**	**–**	**–**	**+**
**CSF WBC/mm^3^**	675		610	343	165	65	42	8	8	5	10	120	20	5	704
**CSF lymphocyte %**	60		40	86	40	65	80					70			82
**CSF protein**	1.7		1.4	2.0	0.9	0.5	0.8	0.3	0.9	0.4	1.1	0.7	0.3	0.8	1.1

+ and – symbols indicate present and absent, respectively.

§ Other symptoms: 006 and 008 had lymphadenopathy; 011 had papilloedema; 031 had an abnormal abdominal exam.

† These patients developed encephalitis signs after admission and CSF was collected at that time.

### Laboratory Findings on Admission

CSF was collected from fourteen AME patients within the first few days of admission (Table 1). CSF was clear in thirteen (93%), but pleocytosis was present in nine (64%) and was particularly pronounced in one patient with cloudy CSF. Lymphocytes were predominant in most of those with CSF with pleocytosis. CSF protein was elevated in ten (71%) and CSF:sera glucose ratio was low in 2 (14%). Opening pressure was recorded for twelve and was normal. All CSF samples were bacterial culture and measles virus RT-PCR negative. Measles virus isolation was not attempted. Throat swabs collected from patient 007 on the first three days of admission (5–7 days after fever onset) were positive for measles virus RNA by RT-PCR.

Neutrophilia (>8000 neutrophils/µl of blood) was present on admission for five (33%) of the AME patients but none of the controls (p = 0.044). Most AME patients and controls were lymphopenic at admission, and there was no significant difference between cases and controls assessed on equivalent illness days. Encephalitis patients had lower CD4 (218/µl, p = 0.029) and CD8 (200/µl, p = 0.012) T cell counts than controls.

**Figure 2:**
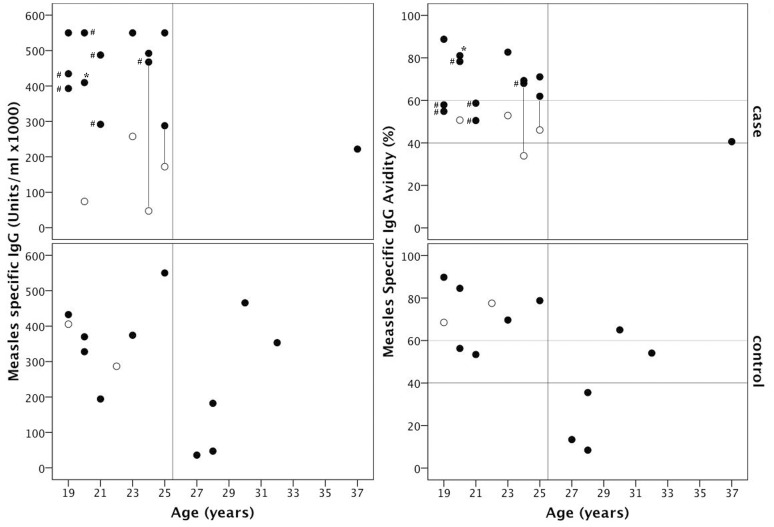
Relationship between age and serum concentration and avidity of measles-reactive IgG in AME patients and controls. Results are shown for 15 AME patients (top panels) and 13 controls (bottom panels). Results are categorized as being tested before (○) or from day 7 since fever onset (•) because titers increased from day 7. Results for tests done before and after day 7 since fever for two AME patients are joined by a line. One patient tested more than 19 days after fever onset is indicated (*). Sera that were tested after administration of IVIG are also indicated (#). Vertical lines differentiate patients born before and after measles vaccine was widely available in Vietnam. Horizontal lines in panel B indicate avidity cut-offs for identifying secondary (upper) and primary (lower) type antibody responses with intermediate levels being indeterminate.

We examined antibodies specific for VGKC-complex, GAD and NMDA receptors because they are now mainstream for the investigation of possible immune-mediated forms of encephalitis [34]. Two AME patients and one control had antibodies specific for the VGKC-complex (Figure S1). The VGKC-complex antibody titer was particularly high in one patient (patient 014, 900 pM), and declined to 609 pM at the 3 month follow-up assessment. Tests for all remaining neuronal antibodies were negative (data not shown). MOG antibody test scores were negative (0–0.5) in all patient sera whereas all four positive controls were strongly positive (2.5–3.5). ELISA to determine measles-reactive IgG concentration and avidity was performed on serum samples collected 5 to 19 days after fever onset, with the exception of one sample collected 25 days after fever onset from an AME patient that presented late (Figure 2). Measles-reactive IgG was not detected in the measles IgM-negative controls that were excluded (data not shown). Measles-reactive IgG concentration and avidity increased substantially between days 5–6 and days 11–12 since fever onset in two AME patients with serial serum assessed (Figure 2). Similarly, measles-reactive IgG concentration was significantly associated with day since fever onset (r = 0.432, p = 0.014). For this reason results were analysed after stratifying by sample collection time, i.e. before or from day 7 since fever onset (Table S1). Measles-reactive IgG concentrations exceeded 100000 units/ml, with the exception of two AME cases tested before day-7 and two controls aged 27 and 28 years (Figure 2). Measles-reactive IgG concentration was not associated with age (r = −0.241, p = 0.183) whereas IgG avidity decreased significantly with increasing age (r = −0.479, p = 0.006, Figure 2). IgG avidity was below 40%, consistent with a primary antibody response, in three controls, all aged more that 25 years. Avidity in the remaining patients was suggestive of secondary antibody responses or was indeterminate. The association between avidity and age was maintained when days since fever onset was included in linear regression (r = −0.453, p = 0.010). There was no observed relationship between measles-reactive IgG concentration and AME, even when comparing patients assessed at similar times since fever onset and in similar age groups but sample sizes were small (Table S1).

### MRI Findings

**Figure 3:**
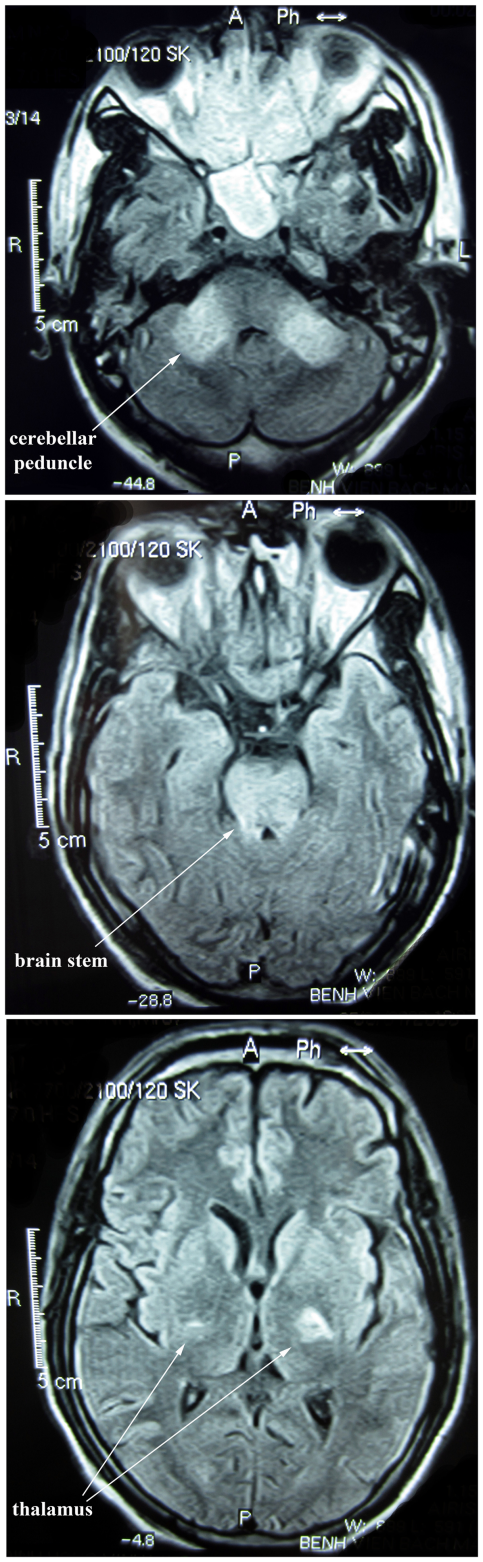
Brain MRI images for an AME patient scanned 9 days after the onset of confusion. FLAIR images show high-intensity signals in the cerebellar peduncle, brain stem and thalamus.

Brain MRI, with T2-weighted and FLAIR sequences, was performed on 13 AME patients in the first few days of admission, and five had a second scan 3 to 11 days later. If abnormality was found gadolinium contrast-enhanced imaging was also performed. MRI images appeared normal for five patients, and in four the only detectable abnormality was paranasal sinus inflammation. The remaining four patients (001, 002, 004, 005) had abnormal MRI images. Their scans were performed 9–15 days after confusion began, whereas earlier scans from two of these patients were normal. Scans were performed during the first week of confusion in most other patients. Abnormalities included bilateral hyperintense lesions on T2-weighted and FLAIR images in the brain-stem, pons, cerebellar peduncle (Figure 3), thalamus, outer temporal lobe and temporal operculum. Slight irregular contrast enhancement was detected in one patient. Abnormalities were not detected in the corpus callosum or hypothalamus.

### Progression, Complications, Treatment and Outcome

**Figure 4:**
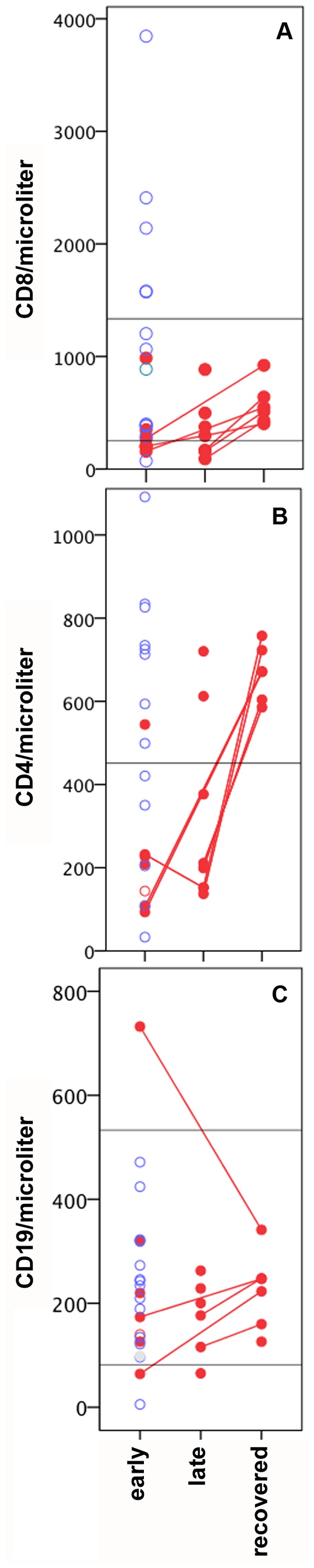
Lymphocyte subset counts in AME patients versus controls. Results for CD8 (A), CD4 (B) and CD19 (C) counts during early illness (day 5–13) are shown for 13 controls (blue circles) and 6 AME patients (red filled circles). Results are also shown for AME patients in late illness (n = 8) and following recovery (n = 6) with lines connecting multiple time points for the same patient. Horizontal lines represent the lower +/− upper limits of the normal range.

The duration and severity of AME varied considerably. Confusion lasted 2–36 (median 12) days (Figure 1), and patients were discharged 14–62 (median 24) days after fever started (Table S2), and 6–55 (median18) days after admission. Controls were discharged 6–13 (median 9) days after fever started and 3–8 (median 4) days after admission (data not shown). GCS scores returned to normal within the first week of admission in nine patients but not until at least the third week of admission in five patients (003,004,005,008 and 031, Figure S2b). Four had persistent fever (Figure S2a, 003, 007, 012 and 031). Limited CSF investigations indicate that pleocytosis (Figure S2c) and proteinorrhachia (Figure S2d) resolved within 1 week of admission with the exception of patient 004. Neurological symptoms became severe at some stage of illness in six (40%) AME patients (Figure S2b), of which four required intubation for 4 to 21 days. Four patients had suspected secondary infections including ventilator associated pneumonia, and tuberculous meningitis (patient 004) indicated by high CSF protein (Figure S2d) and low CSF:serum glucose ratio. During the first two weeks of illness most AME patients but few controls had T cell lymphopenia (Figure 4a&b) and T cell counts were significantly lower in AME patients whereas CD19/B cell counts were normal (Figure 4c). T cell lymphopenia was not detected in AME patients assessed following recovery (Figure 4a&b).

Most AME patients were given dexamethasone within the first 4 days of admission for varying durations (Figure 1), 64% were given intravenous immunoglobulin (IVIG 5000 mg/day, EGIS pharmaceuticals, Budapest, Hungary) within 6 days of admission (Figure 1), and 93% were given piracetam, a derivative of GABA, 0–13 days after admission (Table S2). Antibiotics were given to 79% of AME patients 0–15 days after admission (Table S2). Ceftriaxone, an empiric treatment for meningitis in Vietnam, was given to 40%. Confusion resolved 1–37 (median 6) days after dexamethasone commencement. All AME patients eventually recovered and nine had fully recovered at discharge (Table S2). One was transferred to another hospital and then recovered. Patient 004 had a GCS verbal score of 3 at discharge but had recovered when assessed 3 months later. Patients 003, 005 and 008 had moderate sequelae at discharge including an inability to walk 5 meters but all patients made a full recovery within 1 year of discharge (Table S2).

## Discussion

We describe fifteen young adults that presented with measles and typical acute encephalitis symptoms, including acute onset of fever, headache, confusion and CSF pleocytosis of predominantly mononuclear cells [35]. A number of patients also had MRI findings consistent with descriptions of AME elsewhere [23], [36]–[37]. In particular Lu *et al* report frequent detection of bilateral and symmetrical lesions with poorly defined margins in the midbrain (94%) and pons (71%) [36]. The majority reported being given a single dose of measles vaccine at around 1 year of age. Although recall of childhood vaccination is unreliable, most patients were born after measles vaccine was introduced, and had measles-reactive IgG at high titer and high or intermediate avidity whereas several patients born before this time had low titers and avidity. We also observed a significant trend for avidity to decline with increasing age as may be expected if patients born in the post-vaccine era had been vaccinated at a similar young age and then experienced a gradual decline in avidity with age. The alternative possibility, that primary measles IgG responses are less vigorous with increasing age seems unlikely in our patient group, who were all aged less than 40 years. Secondary measles vaccine failure (infection attributable to a gradual decline in vaccine induced neutralizing antibodies [38]) is not uncommon following single dose vaccination but the development of CNS complications in measles infection following secondary vaccine failure has rarely been reported [2], [38]. We are aware of only one other report that presents two patients with CNS involvement due to secondary vaccination failure [32].

AME started around four days after rash similar to studies elsewhere [13], [15]. Most studies using functional virus detection assays indicate that virus is cleared by this time [39]–[40], and this has been the main basis for proposals that AME is a post-infectious, autoimmune phenomenon [12]–[13]. However, recent studies have detected viral RNA in throat swabs or blood ≥14 days after rash onset in 14% of measles patients [41], and inadequate control of CNS measles virus infection leads to encephalitis in mouse models [11]. Therefore, a direct contribution of infection or of the anti-viral immune response cannot be ruled out as a factor in human AME pathology. The other source of evidence for an autoimmune pathology is the detection of a modest proliferative T-cell response to myelin basic protein in some individuals. Several mechanisms have been proposed to account for the induction of myelin-reactive T cells including mimicry between viral and self antigens [42], blood brain barrier breakdown allowing access to antigens in immune privileged site [43]–[44], and increased production of auto-reactive cells during recovery from lymphopenia [45]. Regulatory T cells play a key role in maintaining peripheral immunologic self-tolerance and preventing autoimmunity [46], and are induced following measles infection [47]–[49]. We found lower CD4 and CD8 cell counts in AME patients compared to measles control patients, which could reflect the preferential induction of Th2 cytokines and blockade of T cell proliferation described elsewhere [15]. Autoantibody production is not uncommon following infection with a range of viruses, and has been associated with auto-immune syndromes and a breakdown of tolerance [50]. VGKC complex auto-antibodies were detected in two AME patients and one control. The level was high in one patient and in this case may represent a specific immune reaction. These potentially pathogenic antibodies have been detected in some patients with limbic encephalitis and in patients with other seizure-related disorders, including epilepsy [29] and faciobrachiodystonic seizures [51]. However, as these high levels were detected in only one patient, it is unlikely that the VGKC complex antibodies play a major role in disease pathogenesis in the majority of the measles encephalitis patients. As discussed earlier, the contribution of myelin-reactive antibodies has been controversial [17] and we did not detect antibodies reactive with MOG in the patients studied here.

Recent guidelines for AME recommend using high dose steroids with IVIG used as a second line treatment on the basis of reports of rapid recovery following treatment [19], [52]–[54]. In the current study, recovery times were variable following prompt treatment with immune-modulators, however all recovered fully whereas historically around 15% die and 30% have long-term sequelae [13], [18]. Randomized trials are needed to establish if these treatments are safe and beneficial.

This study has several limitations. All presenting AME patients were recruited into this study but the number was small as was the number of times that patients were examined, particularly by MRI. Serial investigation of peripheral blood cells to determine virus clearance time would have been valuable as an indirect indication of whether virus could be present in the brain at the time of encephalitis. In six patients measles-reactive IgG was assessed after administering IVIG, which is likely to contain measles-reactive IgG. However, measles-reactive IgG results were not clearly different in these patients (data not shown), and controls did not receive IVIG but trends for measles-reactive IgG were similar.

The occurrence of a large outbreak of measles in young adults and the development of AME in some who appear to have received a single dose of measles containing vaccine during infancy demonstrates the importance of maintaining high coverage with a two-dose measles vaccination schedule. T cell counts were relatively low in AME patients and it will be important to determine whether this could affect control of virus replication or regulation of autoimmunity.

## Supporting Information

Figure S1 **Voltage gated potassium channel-complex specific antibody in serum from 15 AME patients (cases) and 13 controls.** The horizontal line indicates the cut-off for positivity, i.e. 3SD above the mean of healthy controls (100 pM).
(TIF)

Figure S2 **Changes in clinical and laboratory findings during admission.** Results for 15 AME patients are shown in all panels. AME patients with severe illness are indicated in filled red circles and those with moderate or mild illness are shown in open orange circles. Horizontal lines in panel B indicate the GCS score division between severe, moderate and mild CNS disease and the asterisks indicate that the patient was intubated.
(TIF)

Table S1 **Measles-reactive IgG titer and avidity by age, severity and days since fever onset.**
(DOCX)

Table S2 **Treatment and outcome of AME.**
(DOCX)
