# Supplementary figures and images for: Acute Measles Encephalitis in Partially Vaccinated Adults
Source: PLoS One. 2013 Aug 13;8(8):e71671. doi: 10.1371/journal.pone.0071671 (PMC3742472; doi:10.1371/journal.pone.0071671)

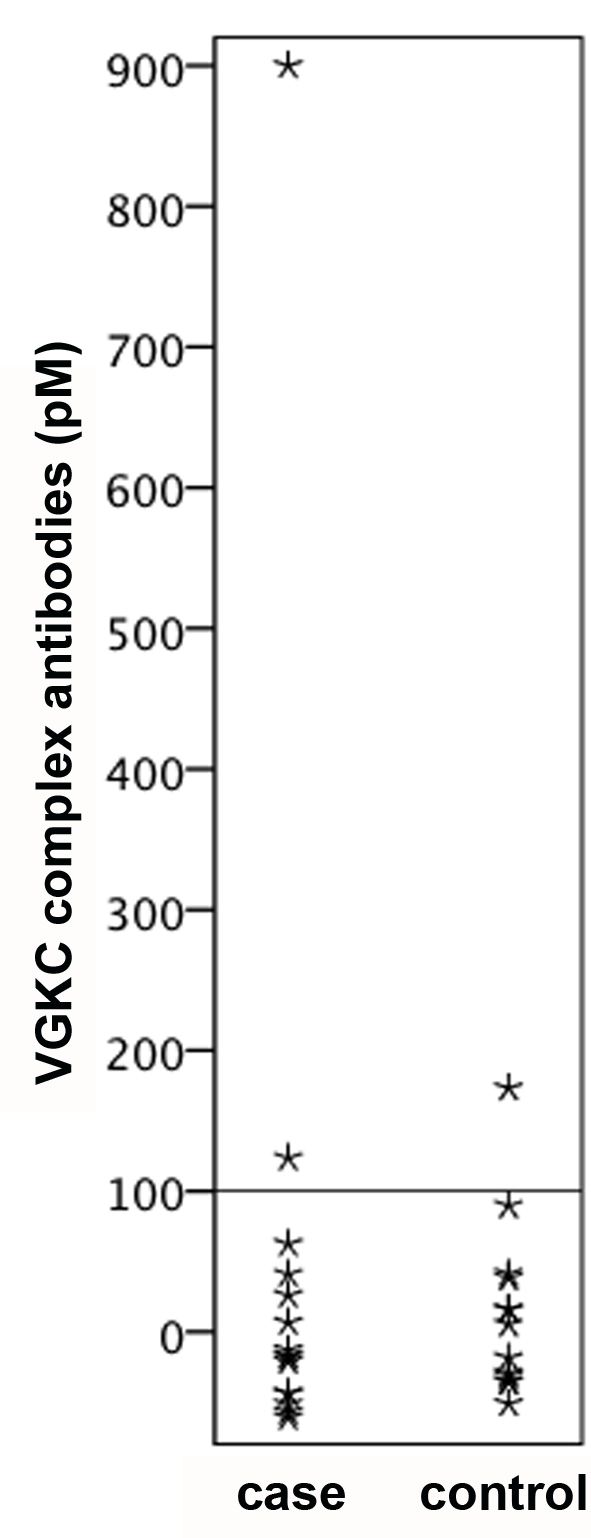

Supplement: Figure S1 — Voltage gated potassium channel-complex specific antibody in serum from 15 AME patients (cases) and 13 controls. The horizontal line indicates the cut-off for positivity, i.e. 3SD above the mean of healthy controls (100 pM). (TIF) [file pone.0071671.s001.tif]

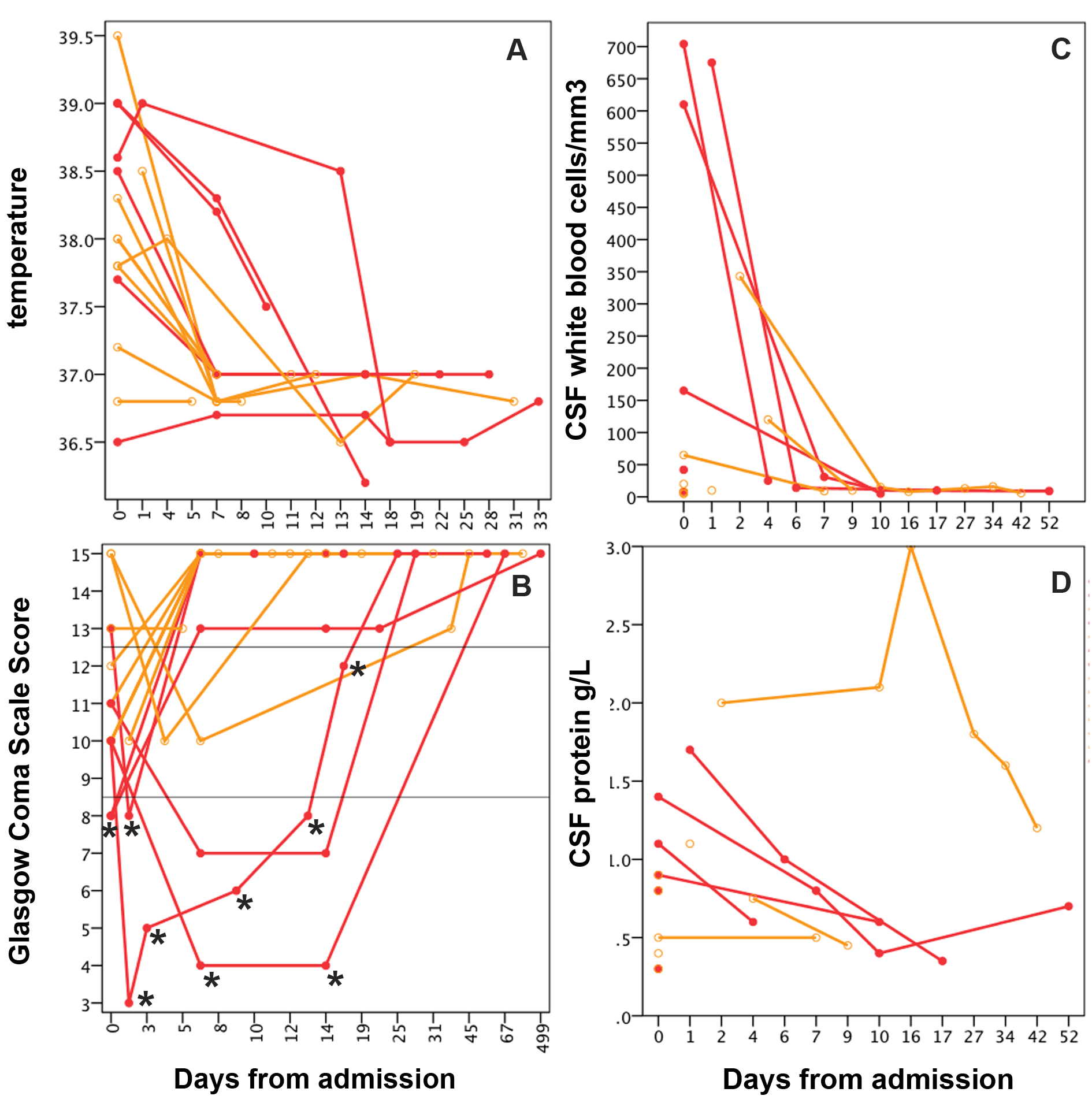

Supplement: Figure S2 — Changes in clinical and laboratory findings during admission. Results for 15 AME patients are shown in all panels. AME patients with severe illness are indicated in filled red circles and those with moderate or mild illness are shown in open orange circles. Horizontal lines in panel B indicate the GCS score division between severe, moderate and mild CNS disease and the asterisks indicate that the patient was intubated. (TIF) [file pone.0071671.s002.tif]
