# Supplementary material for: Acute Measles Encephalitis in Partially Vaccinated Adults
Source: PLoS One. 2013 Aug 13;8(8):e71671. doi: 10.1371/journal.pone.0071671 (PMC3742472; doi:10.1371/journal.pone.0071671)
Supplement: Table S1 — Measles-reactive IgG titer and avidity by age, severity and days since fever onset. (DOCX) [file pone.0071671.s003.docx]

**Table S1. Measles-reactive IgG titer and avidity by age, severity and days since fever onset**

| **Age** | **Severity** | **Days since fever onset** | **N** | **Avidity %** | **U/ml x 10^5^** |
| --- | --- | --- | --- | --- | --- |
|  |  |  |  | *Individual values* or median (range) | |
| ≤ 25 | severe AME | 5-6 | 1 | *53* | *2.57* |
|  |  | >=7 | 4 | 58 (50-81) | 4.01 (2.92-4.87) |
|  | mild AME | 5-6 | 3 | 46 (34, 51) | 0.74 (0.47-1.72) |
|  |  | >=7 | 8 | 70 (55-89) | 5.21 (2.88-5.50) |
|  | control | 5-6 | 2 | *69,78* | *2.87, 4.06* |
|  |  | ≥7 | 6 | 74 (53-90) | 3.72 (1.94-5.50) |
| >25* | severe AME | ≥7 | 1 | *41* | *2.25* |
|  | control | ≥7 | 5 | 36 (8-65) | 1.82 (0.36-4.66) |

*** None of the patients with mild AME were > 25 years and none of the patients aged >25 years were tested before day 7**
