# Supplementary material for: Acute Measles Encephalitis in Partially Vaccinated Adults
Source: PLoS One. 2013 Aug 13;8(8):e71671. doi: 10.1371/journal.pone.0071671 (PMC3742472; doi:10.1371/journal.pone.0071671)
Supplement: Table S2 — Treatment and outcome of AME. (DOCX) [file pone.0071671.s004.docx]

**Table S2. Treatment and outcome of AME**

| **Patient ID** | 001 | 002 | 003 | 004 | 005 | 006 | 007 | 008 | 009 | 010 | 011 | 012 | 013 | 014 | 031 |
| --- | --- | --- | --- | --- | --- | --- | --- | --- | --- | --- | --- | --- | --- | --- | --- |
| **Patient weight (kg)** | 59 | 55 | 60 | 55 | 40 | 53 | 60 | 60 | 40 | 50 | 42 | 45 | 51 | 45 | 53 |
| **Dexamethasone mg/day** | 8 | 24 | 16 | 16 | 16 | 16 | 16 | 16 | 16 | 16 | 32 | 24 |  | 32 | 24 |
| **Piracetam^✝^** | 16^3^ | 0 | 31^8^ | 33^13^ | 19^7^ | 9^6^ | 11 | 29 | 2 | 10 | 5^1^ | 15^5^ |  | 3^1^ | 38^9^ |
| **Antibiotics^✝^** | 17 |  | 53 | 28^6^ | 15 | 23 | 4 | 15^15^ |  |  | 4^1^ |  |  |  | 48 |
| **Number of antibiotics** | 1 | 1 | 11 | 6 | 1 | 3 | 2 | 3 | 3 | 0 | 1 | 0 |  | 0 | 6 |
| **Other medications^¶^** | - | - | + | + | + | + | - | + | + | - | + | - |  | - | - |
| **Onset to discharge (days)** | 25 | 17 | 73 | 50 | 29 | 37 | 18 | 44 | 13 | 24 | 18 | 22 | 16 | 15 | 54 |
| **Discharge GCS** | 15 | 15 | 15 | 13 | 13 | 15 | 15 | 15 | 15 | 13 | 15 | 15 | 15 | 15 | 15 |
| **Discharge clinical score** ^§^ | **5** | 5 | 3 | 5 | 3a | 5 | 5 |  | 5 | 2 | 5 | 5 | 5 | 5 | 3a |
| **Follow-up clinical score** ^§^ |  | 5 | 3 | 5 | 5 | 5 |  | 5 | 5 | 5 | 5 |  |  | 5 |  |

✝ Results are shown as the number of days of treatment with values in superscript being the day of admission that treatment commenced if not at day 0

¶ Other medications: 003 alpha chymotrypsin & muxystin (acetyl cytsteine) on admission, pantoprazole day 2, anti emetic day 4, adrenalin day 7; 004 Bidilucil, tuberculosis chemotherapy from day 18; 005,006 &008 Bidilucil; 009 pantoprazole; 011 adrenalin

§ Clinical score: 5 = Full recovery; 4 = Minor sequelae not affecting function/significant personality change/seizures controlled by anticonvulsants; 3 = Moderate sequelae mildly affecting function but not independent living/uncontrollable seizures; 3a or 3b = unable or able to walk 5 meters independently; 2 = Severe sequelae, making patient dependent; 1 = Death
